# Phosphonates as Unique Components of Plant Seeds—A Promising Approach to Use Phosphorus Profiles in Plant Chemotaxonomy

**DOI:** 10.3390/ijms222111501

**Published:** 2021-10-25

**Authors:** Dorota Wieczorek, Beata Żyszka-Haberecht, Anna Kafka, Jacek Lipok

**Affiliations:** Department of Pharmacy and Ecological Chemistry, Institute of Chemistry, University of Opole, Oleska 48, 45-052 Opole, Poland; beata.zyszka-haberecht@uni.opole.pl (B.Ż.-H.); anna.kafka@uni.opole.pl (A.K.); jacek.lipok@uni.opole.pl (J.L.)

**Keywords:** phosphorus, phosphorus profile, ^31^P NMR, seeds, phosphonates

## Abstract

Phosphorus is one of the most important elements essential for all living beings. Plants accumulate and store phosphorous in various forms that have diverse physiological and biochemical functions. In this study, we determine and then examine the phosphorus profiles of seeds of plants belonging to different taxa based on extractable inorganic phosphates and organic forms of phosphorus. We paid particular attention to the presence of natural phosphonates in the tested materials. The inorganic phosphates were determined colorimetrically, whereas phosphorus profiles were created by using ^31^P NMR spectroscopy. Our study on phosphorus profiles revealed that the obtainedsets of data vary significantly among the representatives of different taxa and were somehow specific for families of plants. It should be emphasised that the measurements obtained using ^31^P NMR spectroscopy undoubtedly confirmed—for the first time—the presence of phosphonates among the natural components of plant seeds. Hence, the classification of plants considering the phosphorus profiles, including the presence of phosphonates, may be a new additional chemotaxonomic feature.

## 1. Introduction

Phosphorus (P) is one of the most important nonmetallic elements essential for all organisms. Its presence in a living system is indispensable for facilitating diverse metabolic processes such as energy transfer, signal transduction, or enzyme regulation [1]. In cells, it is generally found in the form of inorganic phosphate (Pi) and its derivatives. These chemical connections are mainly esters where the residue of orthophosphoric acid is bound through oxygen atoms to organic molecules such as lipids, sugars, or proteins [2]. The main pools for esterified P are nucleic acids (DNA and RNA), phosphoproteins, phospholipids, sugar phosphates, and energy-rich phosphorylated nucleotides (e.g., adenosine triphosphate) [3]. It is worth emphasizing that, apart from the most common phosphates, natural derivatives of phosphonic acid, characterised by the presence of direct covalent carbon to phosphorus bonds, may also exist in living cells. Despite the fact that geological studies have indicated the occurrence of phosphonates on Earth for billions of years, the existence of natural phosphonic compounds as a component of living beings was proven only in the second half of the 20th century [4], when Horiguchii and Kandatsu isolated 2-aminoethylphosphonic acid (2-AEP, ciliatine) from flagellates of rumen protozoa [5]. Since then, 2-AEP has been shown to occur in both free and bound forms in many groups of organisms, such as molluscs, lower fungal insects, and mammals [6]. The discovery of ciliatine initiated intensive studies on the distribution of phosphonates in nature. In the following years, Hendlin et al. isolated fosfomycin (1,2-epoxy-propanephosphonic acid) from actinomycetes belonging to the *Streptomyces fradiae* species [7]. Fosfomycin was the first phosphonate discovered to have antibiotic properties. Its activity relies on blocking bacterial cell wall synthesis by inhibiting peptidoglycan formation [8,9]. Another known phosphonate is bialaphos, which is produced by *Streptomyces viridochromogenes* and *Streptomyces hygroscopicus*. This substance possesses phytotoxic activity and is widely used as a nonselective herbicide. Chemically, bialaphos is a tripeptide consisting of two molecules of L-alanine and phosphinothricin, which is crucial for its activity. Phosphinothricin is a phosphonic analogue of glutamine that acts as a competitive inhibitor of glutamine synthetase, an enzyme involved in the metabolism of nitrogen in bacteria and plants [6]. Currently, except for the abovementioned phosphonic derivatives, several dozen naturally occurring phosphonic derivatives are known [10,11]. There are diverse hypotheses regarding their metabolic function in organisms. According to these hypotheses, phosphonates may be involved in cell signalling, protect the cell from enzymatic degradation, act as antimetabolites, or constitute a cell phosphorus reservoir [4,12].

Data regarding naturally occurring phosphonate compounds relate mainly to structures isolated from microorganisms (bacteria, cyanobacteria, fungi) and animals [6]. Although qualitative and quantitative research on phosphorus compounds in plant organs and tissues has been described many times [13,14,15], the presence of phosphonates in plant materials has been confirmed only sporadically. The only reports of phosphonic compounds present in plants come from the 1980s. In 1984, Moschidis isolated phosphonolipids from apricot kernels [16]. Five years later, Mukhamedova et al. determined the phosphonolipids in kenaf seeds [17]. However, the respective results presented in both mentioned reports were based only on TLC separations and IR spectroscopy in the case of Moschidis, without any real phosphonolipid compounds used as standards for those determinations. Since then, there has been no new information confirming the presence of phosphonates in any organ at any stage of plant growth. In that case, the question remains whether the presence of phosphonates is a rare, individual feature of some plant species or whether these compounds are commonly abundant in the plant kingdom. Therefore, the aim of our work was to examine the phosphorus profiles of seeds of plants belonging to different taxa. Confirmation of the presence of these substances as the specific components of phosphorus profiles may lead to a broadening of plant taxonomy using new parameters, as well as to an enrichment of the knowledge on the biological functions of phosphonic compounds in plants.

## 2. Results

### 2.1. Inorganic Phosphates (Pi) in Plant Seeds

**Figure 1:**
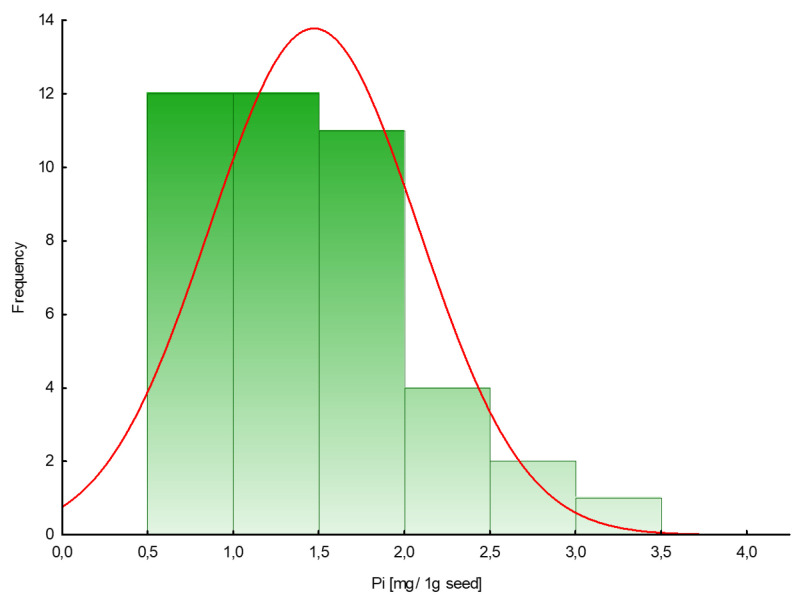
Histogram of the frequency distribution of the amount of phosphate in seeds (mg/1 g seed).

The amount of phosphate extracted in samples of plant seeds is presented in Table 1. To measure free phosphate in the tested plant seeds, a colorimetric assay with malachite green acid dye was used. The phosphate content in seeds varied from 0.6 to 3.4 mg/1 g seed (mean value 1.5), with lower values for dill ‘Szmaragd’ and parsley ‘Alba’ seeds and higher values for pepper ‘Kasia’ seeds. Figure 1 shows the frequency distribution of a given amount of inorganic phosphates (mg) in 1 g of seeds.

### 2.2. Phosphorus Speciation of Plant Seeds by ^31^P NMR

**Figure 2:**
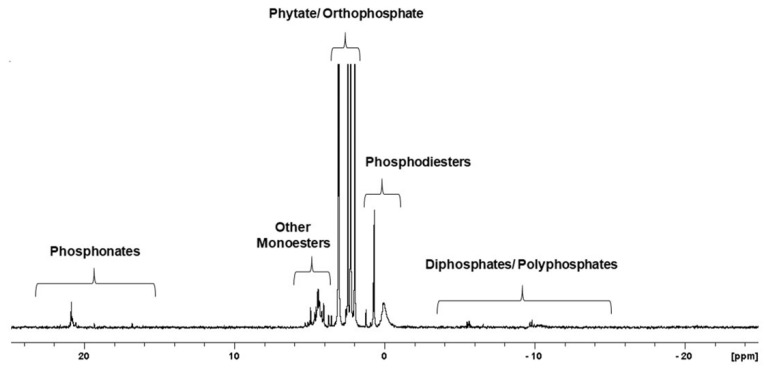
^31^P NMR spectrum showing the range of P compounds detected in the samples for this study. This spectrum shows the phosphorus profile of the neutralised extract of cucumber ‘Aladyn F1’ (CUC1).

^31^P NMR spectroscopy is a suitable technique for the detailed characterization of all P species, including inorganic and organic P forms, in tested samples. Figure 2 presents the detection range of individual forms of phosphorus on the ^31^P NMR spectra of the neutralised extract of cucumber ‘Aladyn F1’ (CUC1).

The ^31^P NMR spectrum of the phosphorus compounds found in the neutralised seed extract from cucumber, as an example (Figure 2), reflects the phosphorus profile of those seeds. The presented spectrum comprises several groups of resonance peaks located between -20 and 25 ppm chemical shifts that correspond to different groups of phosphorus compounds. These groups were identified on the basis of the predicted chemical shift characteristics for individual P species at neutral pH [18]. Generally, the presence of pyro- and polyphosphatesis confirmed by the peaks lying between −15 and −3 ppm. In the range of −1 to 1.5 ppm, orthophosphate diesters are presented. Phosphodiester bands include nucleic acid residues, mainly DNA (broad signal near 0 ppm) and intact phospholipids. Signals between 1.5 and 3.7 ppm are attributable to phytates and orthophosphates. The resonance signals of phytates are relatively easy to recognise due to their characteristic peak pattern resulting from the isomeric forms with different positions of the dissociated phosphate group bound to the inositol ring. Other monoesters, such as glucose 1-phosphate, were detected at 3.7–6 ppm. The last separated group of organophosphorus compounds, natural phosphonates, generated peaks between 15 and 23 ppm.

Quantification of the particular groups of phosphorus compounds was performed by spectral deconvolution analysis based on chemical shifts and peak areas. The P species found in the neutralised seed extracts and the relative percentage of individual P species based on the total NMR area are listed in Table 1.

**Figure 3:**
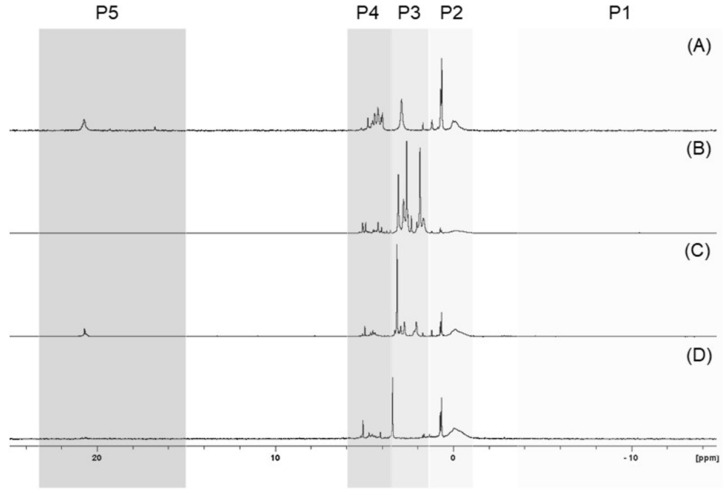
Stacked and normalised ^31^P NMR spectra of chosen plant seed extracts: carrot ‘Dolanka’ (**A**), green lentil sprouting seeds (**B**), broccoli sprouting seeds (**C**), and sunflower sprouting seeds (**D**), with indications of profile clusters P1–P4. The spectra show areas corresponding to polyphosphates/diphosphates (P1), phosphodiesters (P2), phytates/orthophosphates (P3), other monoesters (P4), and phosphonates (P5).

On the basis of the results obtained, it can be concluded that the tested seeds possess differential phosphorus profiles, although some similarities can also be noted. For example, the ^31^P NMR spectra of the P species found in the neutralised seed extracts representing four clusters are shown in Figure 3.

Phosphorus in seeds occurs almost exclusively in the organic form of phytates and in the inorganic form of orthophosphates (P3 band) as the major P-containing species in most of the studied seeds, and its amount was established from 41.1% for FAB6 seeds to 92.3% for CUC3 seeds with respect to total neutralised extractable P. In the seeds, the dominant forms of phosphorus were mainly phosphodiesters (P2) (from 38.6% for API1 to 86.3% for FAB2), and other monoesters (P4) (from 27.3% for API6 to 85.4% for FAB1) were assigned. Phosphorus forms recorded in the P1 band are either not present in the seeds at all or may contribute little to the overall P profile. To date, phosphonates have not been included in the phosphorus profile of seeds. However, our findings suggest that their participation cannot be underestimated. The proportion of phosphonates ranged from 0.1% to 9.7% of the total neutralised extractable P. It should be noted that the percentage content of particular groups of P compounds was designed based on the area of the peaks presented in the ^31^P spectra. The sum of areas of the peaks occurring in the analysed range of the ^31^P NMR spectra, divided in P1–P5 regions, was expressed as a percent to the sum of all the peak areas presented on a spectra.

**Figure 4:**
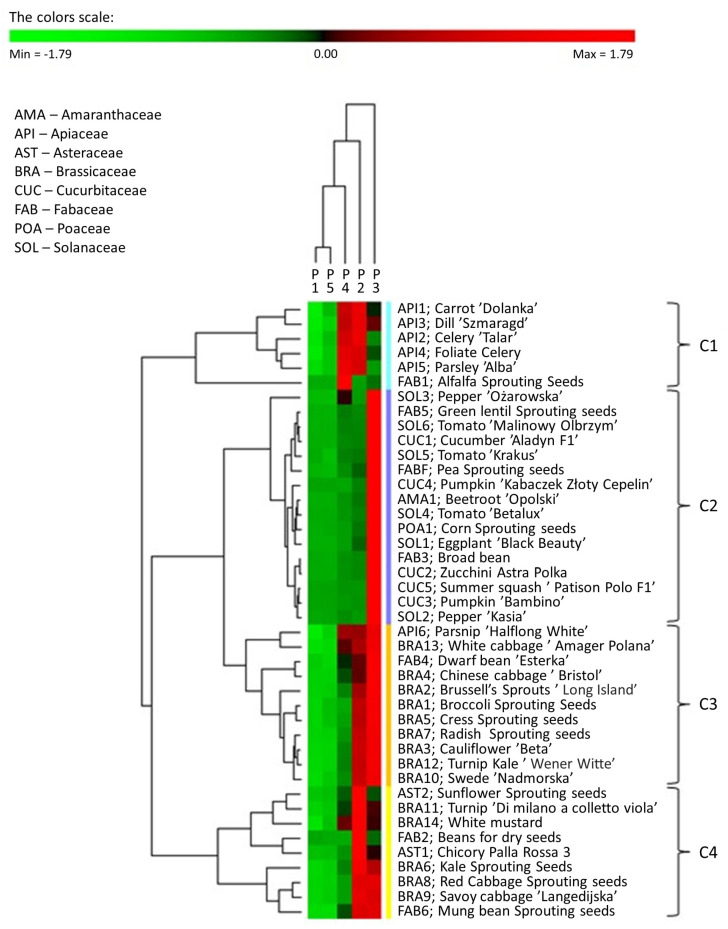
MHCA of the phosphorus profiles of tested plant seeds obtained with PermutMatrix program v.1.9.4 (LIRMM, France). Z-transformation of the dataset, Euclidean correlations, and the complete linkage method were used to perform clustering. Column dendrogram: types of phosphorus species. Seeds shown as assignments/symbols are indicated on the right. Coloured bars (clusters C1–C4) on the right of the heatmap mark distinct major branches in the clustering tree grouping seeds with similar expression patterns. The colour scale indicates the percentage value (light red indicates a higher percentage value, light green indicates lower phosphorus species values).

To summarise and show possible correlations within the phosphorus varieties of the examined seeds of plants, the ^31^P NMR phosphorus profiles are presented as a heat map with dendrograms (Figure 4).

To correlate the parameters of various types of plant seeds, a matrix hierarchical cluster analysis (MHCA) was performed on the 42 selected kinds of seeds using the content of individual phosphorus species variables. To express the mentioned correlations and link analyses between the phosphorus profiles and the seeds, an object and feature grouping procedure was used, and its results were assembled into a colour map. A z-value transformation was used for all variables. The clustering of the selected seeds on the heat map was performed using a hierarchical clustering algorithm that uses a complete linkage method and a Euclidean correlation coefficient. Light green indicates phosphorus species that occur in relatively low percentages in the phosphorus profiles of seeds, whereas bright red indicates phosphorus species that are present in high percentages.

The analysis of the dendrogram shows that the tested seeds can be divided into four major clusters. The first group (cluster 1) consists of the seeds of six plant species belonging to one family, the celery family (API), with the exception of parsnip ‘Halflong White’(API6). The phosphorus profile of seeds assigned to cluster C1 consisted mainly of phosphodiesters (P2) and other monoesters (P4), each accounting for 32% to 45%. A feature of the seeds belonging to cluster C1 is that phytates and orthophosphates (P3) were not predominant forms of phosphorus. Polyphosphates/diphosphates (P1) were detected only in the API4 and API5 seeds at a low percentage (0.2–0.7%) of the total neutralised extractable P. Surprisingly, the third most abundant P species were phosphonates. In particular, the highest percentage of phosphonate was found in the parsley ‘Alba’ seed (API5) (9.7% related to the total neutralised extractable P), while the lowest was found in the carrot ‘Dolanka’ seed (API1) (4.9% of the total neutralised extractable P). The largest cluster, Cluster 2, includes all seeds belonging to the amaranth (AMA), mustard (CUC), grass (POA), and nightshade (SOL) families and most of the seeds belonging to the legume (FAB) family. The phosphorus profiles of seeds from cluster 2 were dominated by phytates and orthophosphates (P3), which comprised 72.2% of SOL3 seeds and 93.0% of CUC5 seeds (average 83%). The percentages of P2 and P4 in the phosphorus profile of seeds from this cluster were much lower than the corresponding proportions of these phosphorus compounds in cluster 1. The percentage of P2 ranged from 3.3% for CUC5 to 14.8% for FAB7, and the percentage of P4 ranged from 0.2% for CUC4 to 21.1% for SOL3. Most of the seeds assigned to cluster 2 contained no P1 at all. The highest relative percentage of P1 was found in the SOL5 seeds (4.0% of the total neutralised extractable P), while the lowest detectable percentage was found in the SOL6 seeds (0.2% of the total neutralised extractable P). In turn, phosphonates were not identified in four kinds of seeds: FAB3, SOL1, SOL3,and SOL5, and in the case of the AMA1 and CUC2 seeds, the percentage of these compounds was established as 0.1%, whereas it was 2.0% for the CUC1 seeds. Cluster 3 includes most seeds from the Cucurbitaceae (BRA) family and single representatives from the Apiaceae (API) and Fabaceae (FAB) families. The phosphorus in seeds assigned to cluster 3 was principally bound as phytates and orthophosphates as the major P-containing species in all seeds, ranging from 39.9% to 57.3% with respect to the total extractable P. The second most abundant form belonged to P2, and their amount varied from 23.8% for the BRA4 and FAB4 seeds to 37.3% for the BRA3 seeds. The percentage of P4 in the phosphorus profile was also significant, ranging from 8.3% to 27.3%. P1 was only present in these seeds in trace amounts. The content of phosphonates in this group of seeds varied quite significantly from the lowest in the BRA3 seeds (0.8%) to the highest (6.2%) in the API6 seeds. The last cluster, cluster 4, includes seeds belonging to the Asteraceae (AST) family and some seeds from the Cucurbitaceae (CUC) and Fabaceae (FAB) families. In the case of most of these plant species, the dominant form of phosphorus was not P3 (from 12.5% to 44.7% of the total extractable P) but P2 (from 39.9% to 86.3% of the total extractable P). The percentage of phosphonates in the profiles of seeds representing cluster 4 ranged from 0.5% for the FAB6 seeds to 7.4% for the BRA14 seeds. It is worth noting that plants belonging to the Fabaceae family possess the most diverse phosphorus profile, which cannot be unequivocally assigned to a specific cluster.

### 2.3. Natural Phosphonates in Plant Seeds

**Figure 5:**
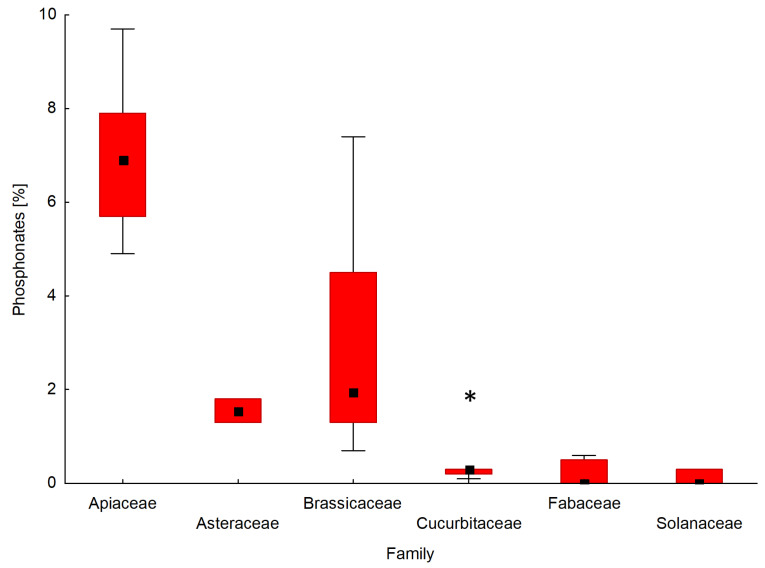
Box and whisker plot presenting the distribution of the content of phosphonates for seeds belonging to different plant families. In the graph, Amaranthaceae and Poaceae were not included because of an insufficient number of analysed plant species. The boxes indicate the median and its confidence limits; whiskers indicate the range of data within the ±1.5 interquartile range; (*) symbols indicate extremely outlier seeds.

Particular attention in this study has been given to the presence of natural phosphonates in the phosphorus profile of the tested seeds. A comparison of the interquartile range and the mean of the content of phosphonates for the seeds of forty-two plant species determined by ^31^P NMR is shown in Figure 5. A box and whisker plot was used to analyse the relationship between the percentage of phosphonates in the phosphorus profile of seeds and a seed’s affiliation with a given plant family.

The seeds of the plants that belong to the API family contained the highest amounts of phosphonates among the tested plant families. The percentages of phosphonates in the phosphorus profile of the seeds of the API family ranged from 4.9% for parsley ‘Alba’ (API5) to 9.7% for carrot ‘Dolanka ’ (API1). In the case of seed plants from the BRA family, phosphonates werepresent in amounts ranging from 0.7% (Chinese cabbage ‘Bristol’; BRA4) to 7.4% (white mustard; BRA14) by weight of the total phosphorus profile. The phosphorus profiles of the seeds from families AST and POA contained phosphonates at the level of 1.3–1.8%. The seeds belonging to families AMA, CUC, FAB, and SOL either contained no phosphonates at all or their amount didnot exceed 1%, with the exception of cucumber ‘Aladyn F1′ from the CUC family (2.0%).

## 3. Discussion

Phosphorus is one of the most important non-metallic elements essential for life. Therefore, in our study we focused first on determining and then examining the phosphorus profiles of the seeds of plants belonging to different taxa based on extractable inorganic phosphates and organic forms of phosphorus. A colorimetric assay was used to measure free phosphate levels in tested plant seeds in the presence of malachite green acid dye. The obtained results are similar to those received for other plant seeds (cumin, fennel, flax, mustard, poppy, sesame) by other investigators and ranged from 1.1 mg/g for cumin to 2.8 mg/g for sesame seeds [19]. According to the calculated frequency distribution, most of the examined seeds contained no more than 2 mg of Pi per 1 g of seeds. 

In comparison other analytical techniques, ^31^P NMR is a valuable tool for the detailed characterisation of all P species, including inorganic and organic P forms, without the need for any complex cleaning and prefractionation procedures. Despite that, phosphorus speciation of seed extracts by ^31^P NMR spectroscopy is poorly described in the literature [15,19]. Our research was based on this technique and enabled the phosphorus profiles of many kinds of seeds belonging to different families to be characterised, while taking into account the proportion of phosphonates. In the characterisation of P species in environmental samples by ^31^P NMR spectroscopy, the samples are commonly made alkaline (pH > 13) to facilitate sample comparison and ease peak identification, but this may cause the hydrolysis or precipitation of some compounds, especially esters. In this study, we analysed P compounds at pH 7.0 ± 0.5 because McDowell and Steward indicated that the distinction between compound classes was best when the sample pH was in this range [18].

All ^31^P NMR spectra showed the presence of orthophosphates, phytates, phosphate monoesters, and diesters, while di- and polyphosphates and phosphonates were detected in only some of the neutralised seed extracts. Phosphorus in the seeds occurred almost exclusively in the organic form of phytates and in the inorganic form of orthophosphates as the major P-containing species in most of the studied seeds. Other dominant forms of phosphorus in the plant seeds were mainly phosphodiesters and other monoesters, whereas diphosphates and polyphosphates were either not present in the seeds at all or contributed little to the overall P profile. On the basis of the results obtained, it can be concluded that the tested seeds possess differential phosphorus profiles, although some similarities can also be noted. A heat map with dendrograms, created based on the ^31^P NMR phosphorus profiles, summarises and shows possible correlations within the phosphorus forms of the examined seeds of plants. In most cases, seeds belonging to the same botanical family were assigned to one cluster; however, some exceptions were observed. For example, plants belonging to the Fabaceae family that possess the most diverse phosphorus profile, which could notbe unequivocally assigned to a specific cluster. The ranges of the abundance of specific forms of phosphorus were relatively wide and often overlapped; however, a statistical approach to the analysis of the phosphorus profiles of the examined seeds confirmed that observed differences were significant and characterised appropriate groups of plant seeds. Therefore, the presence of specific forms of phosphorus in appropriate proportions may be an important chemotaxonomic feature of plants.

Particular attention in this work has been given to the presence of natural phosphonates in the phosphorus profile of the tested seeds. Data regarding naturally occurring phosphonate compounds relate primarily to substances isolated from microorganisms (bacteria, cyanobacteria, fungi) and animals and their biological roles [6,16]. The content of phosphonates in plant materials has been studied only sporadically, and the seeds have not been unequivocally confirmed to date. It is worth noting that, according to endosymbiotic theory, cyanobacteria explain the establishment of primary plastids in plants. Moreover, these photoautotrophic prokaryotes are capable of interacting and establishing long-lived symbiotic associations with representatives among the plant kingdom [20]. These incredible abilities of cyanobacteria in combination with the natural occurrence of phosphonates in cyanobacterial cells may suggest that plants can also synthesise phosphonates. This hypothesis was confirmed by the results of our study. 

To the best of our knowledge, to date, there is no accurate and reliable information in the literature regarding the presence of phosphonates in plant tissues and organs. The presence of phosphonic compounds in the seeds of plants belonging to different taxa has been confirmed for the first time. The only scientific reports on the occurrence of phosphonic compounds in plants were published over 30 years ago. In 1984, Moschidis isolated phosphonolipids from apricot kernels [16], and four years later, Mukhamedova confirmed the presence of phosphonolipids in kenaf seeds [17]. Unfortunately, there were no reliable and unequivocal data confirming those findings included in the cited reports, since no chemical standards or respective analytical methods were used. This may be the reason why some sources state that phosphonic compounds do not occur in plants [21]. Contradictions in the literature make it difficult to understand the current state of knowledge about the presence of phosphonic compounds in plants. Further studies on natural phosphonic compounds have proven that these substances are quite common in cells of evolutionarily older organisms, such as bacteria, fungi, molluscs, and insects. The question is whether phosphonates are only the residue of primary forms of P that occurred in Earth’s early biosphere. There are several hypotheses regarding the role of phosphonates in organisms. According to one of them, the presence of bound phosphonates in external cell membranes may provide protection during contact with parasites [12]. This may be caused by the resistance of C-P bound to an enzymatic degradation product. Moreover, the distribution of phosphonates in the nerve tissues of marine invertebrates suggests that they are involved in the transmission of nerve impulses [4]. It is also believed that due to the chemical similarity of the -PO_3_H_2_ group to the carboxyl group, especially because of the tetrahedral intermediate character of the latter, phosphonates can act as antimetabolites against organisms linked by direct trophic relationships or towards those competing for environmental resources. There are also hypotheses suggesting that organophosphonate compounds may be another reservoir form of phosphorus in the cells of organisms [4,12].

Independent of the fact that the role of phosphonates in plant tissues and organs is still a matter of discussion, our research clearly confirmed the presence of phosphonates among natural components of plant seeds. Moreover, the study on phosphorus profiles of the seeds of plants representing different families of these biota revealed that those profiles vary among the representatives and are somehow specific. The content of phosphonates seems to be similar in particular plant families. This phenomenon may be a useful additional chemotaxonomic feature that may significantly enrich the classification of plants based not only on seed morphology and anatomy, e.g., shapes, surfaces, colour, size, mucilage, wing, hilum, and embryo folding [22]. The classification of plants considering the phosphorus profiles, including the presence of phosphonates, may be a new approach with specific value in terms of resolving cases of taxonomic doubt. However, one should keep in mind that only the analysis of a larger number of seeds from several different plant families can support the relevance of phosphonates for chemotaxonomy. Our study is only first step in this subject.

## 4. Materials and Methods

### 4.1. Tested Plant Seeds

A total of forty-two kinds of seeds representing eight different botanical families of plants, namely, the amaranth family (Amaranthaceae) (AMA), celery family (Apiaceae) (API), sunflower family (Asteraceae) (AST), mustard family (Brassicaceae) (BRA), cucurbit family (Cucurbitaceae) (CUC), legume family (Fabaceae) (FAB), grass family (Poaceae) (POA), and nightshade family (Solanaceae) (SOL), were analysed in this study. The studied plant seeds were mainly purchased from a local plant breeding and seed company located in east-central Poland (PNOS Sp. z o.o., Ożarów Mazowiecki, Poland), except for beetroot (AMA1) and zucchini seeds (CUC2), which were obtained from Garden Seed and Nursery Stock Company in north-central Poland (Torseed S.A., Toruń, Poland). The common and botanical names of the tested seeds, their taxonomic classification by families, the estimated number of seeds per gram, and assigned symbols used in the text are presented in Table 2. All seeds were stored at room temperature in tightly sealed polyethylene bags until use.

### 4.2. Isolation of Phosphorus Compounds from Seeds

Each plant seed was weighed separately, and approximately 5.0 g was ground using a cryogenic mill machine (SPEX 6775 Freezer/Mill; SpexSamplePrep, Metuchen, NJ, USA). The cryogenic mill chills seeds with liquid nitrogen and then pulverises them with a magnetically driven impactor. The milling machine was precooled for 5 min to reach cryogenic temperature before grinding. After that, the seeds were milled for 30 seconds in two grinding cycles at a rate of 15 CPS (cycles per second) with a 1-min intercool. Then, 1.0 g of the ground representative sample (*n* = 3) was mixed with 5 mL of 0.1 M potassium hydroxide (KOH), which was used as an extraction solvent. Ultrasound-assisted extraction (UAE) was performed in an ultrasonic cleaning bath (Cole-Parmer 8891, 42 kHz, 100 W; Vernon Hills, IL, USA) at 25 °C for 30 min. At the end of each sonication period, the whole extract was centrifuged at 5000× *g* for 5 min, and the supernatant was collected. Each sample was extracted three times with a new portion of the solvent. After three extractions, the seeds were washed with 5 mL of distilled water, and the suspension was again centrifuged. All collected supernatants were combined and neutralised with 35% perchloric acid (HClO_4_) (*v*/*v*). The solution pH was adjusted to 7.0 ± 0.5, and, after at least 30 min, the mixture containing the insoluble residue and the resulting precipitate of potassium perchlorate was centrifuged at 5000× *g* for 5 min. The clear supernatant solution was diluted with deionised water to a final volume of 25 mL and then passed through Whatman No. 41 filter paper. The supernatant was divided into two parts: 2 mL was frozen at −28 °C until the determination of Pi content, and the rest was freeze-dried at −50 °C (freeze dryer ChristAlpha 1-2 LD plus) and stored at −28 °C until ^31^P NMR analysis. The entire procedure was performed in triplicate for each sample. All reagents used except malachite green and surfactant (CHAPS) were analytical grade and were purchased from Avantor Performance Materials Poland S.A. (Gliwice, Poland) and Merck (Merck Millipore, Darmstadt, Germany) and used without further purification. The water was treated in a Milli-Q water purification system (Millipore, Bedford, MA, USA).

### 4.3. Determination of Phosphate Content in Seeds

The content of free orthophosphates in all extracts of the tested seeds was measured by spectrophotometric quantitative analysis with the malachite green acid dye procedure, as described by Forlani et al. [23]. Proper sample dilutions in a final volume of 50 μL, obtained with isolation of phosphorus compounds present in the seeds, were supplemented with 1.0 mL of the malachite green–molybdate-acid solution, followed by 0.1 mL of 34% (*w*/*v*) sodium citrate after exactly 1 min. Then, the absorbance of the resulting coloured complex was measured at room temperature at a wavelength of 660 nm against exact blanks at times not longer than 20 min. A standard solution was prepared by dissolving K_2_HPO_4_ in distilled water to prepare different phosphate concentrations, as described above for the tested samples. The calibration curve was linear over a range of 0.01–1 mM (r^2^ = 0.9996). The amount of orthophosphate ions in the tested seed samples was expressed as mg PO_4_^3−^/1 g seed. Reported values are the means ± SD over at least three replicates.

### 4.4. Determination of Phosphorus Profiles of the Seeds

^31^P NMR spectroscopy was carried out to define the phosphorus profiles of the seeds. Each freeze-dried extract obtained after seed extraction was redissolved in a mixture of 0.9 mL of deionised water and 0.1 mL of 0.1 M EDTA in 1 M NaOH. Samples were centrifuged at 13,000× *g* for 5 min to remove particles that might contribute to line broadening during NMR analysis. The pH of all the obtained solutions was adjusted to 7.0 ± 0.5. The supernatant (500 μL) was transferred into a 5mm NMR tube, together with a 1 mM solution of glufosinate as an internal reference standard (δ = 42.68 ppm). ^31^P NMR experiments were performed using a 400 MHz Bruker Avance DRX spectrometer (Bruker, Rheinstetten, Germany) operating at a 161.98 MHz frequency. Data were acquired at 20 ± 1 °C using a 30° pulse, a 1.37-s acquisition time, and a 0.5-s relaxation delay. Broadband proton decoupling and a 20 Hz spin rate were used for all samples. The number of scans was 20,480. The quantification of P species was performed by spectral deconvolution analysis based on chemical shifts and peak areas. The integration of peak areas was calculated on spectra processed with a line broadening of 2 Hz. ^31^P NMR signal assignments were based on literature data [15,24]. The relative P concentrations in the neutralised extracts were estimated based on the total NMR signal area and presented as the percentage of each species using TopSpin version 3.6.2 software. All samples were prepared in triplicate for NMR analyses.

### 4.5. Statistical Analysis

All experiments were carried out in three independent replicates, and data are expressed as the mean ± standard deviation (*n* = 3). Statistical and graphic analyses were performed using STATISTICA 13.1 software (StatSoft, Cracow, Poland), while matrix hierarchical cluster analysis (normalised data, Euclidean distance, complete linkage method) was performed with the PermutMatrix program v. 1.9.4 (LIRMM, Montpellier, France).

## Figures and Tables

**Table 1:** Relative amounts (%) of the major P forms detected in neutralised extracts of tested plant seeds.

Symbol	Common Name	Variety of Seed	Pi Content (mg PO_4_^3−^/g DW Seed)	P1 (%)	P2 (%)	P3 (%)	P4 (%)	P5 (%)
AMA1	Beetroot	Opolski	2.4 ± 0.1	0	10.7	83.3	5.9	0.1
API1	Carrot	Dolanka	0.9 ± 0.0	0	38.6	19.6	32.1	9.7
API2	Celery	Talar	1.1 ± 0.0	0	42.2	14	35.9	7.9
API3	Dill	Szmaragd	0.6 ± 0.0	0	39.5	23.1	31.7	5.7
API4	Foliate celery	Verde Pascal	0.9 ± 0.1	0.7	35.6	18	38.1	7.6
API5	Parsley	Alba	0.6 ± 0.0	0.2	38.7	11.4	44.8	4.9
API6	Parsnip	Halflong White	1.4 ± 0.0	0	26.6	39.9	27.3	6.2
AST1	Chicory	Palla Rossa 3	1.8 ± 0.1	0	68.9	20.9	8.9	1.3
AST2	Sunflower sprouting seeds	-	1.2 ± 0.1	0	65.6	16.7	15.9	1.8
BRA1	Broccoli sprouting Seeds	*-*	0.8 ± 0.0	0	34.8	51.8	8.3	5.1
BRA2	Brussels sprouts	Long Island	1.5 ± 0.0	0	30.7	55.1	13	1.2
BRA3	Cauliflower	Beta	1.5 ± 0.0	0	37.3	48.8	13.1	0.8
BRA4	Chinese cabbage	Bristol	1.6 ± 0.0	0.8	23.8	57.3	17.4	0.7
BRA5	Cress sprouting seeds	-	0.6 ± 0.1	0.1	33.7	52.1	9.6	4.5
BRA6	Kale sprouting Seeds, fringed cabbage	-	1.2 ± 0.0	0	51	33.3	13.4	2.3
BRA7	Radish sprouting seeds	-	1.0 ± 0.1	0.4	36.6	50.6	11.1	1.3
BRA8	Red cabbage sprouting seeds	-	1.2 ± 0.1	0.1	43.8	44.7	9.9	1.5
BRA9	Savoy cabbage	Langedijska	1.4 ± 0.0	0	46.2	42.1	9.8	1.9
BRA10	Swede (rutabaga)	Nadmorska	0.7 ± 0.1	0.3	35.8	46.5	12.8	4.6
BRA11	Turnip	Di milano a collettoviola	1.1 ± 0.0	0	56.8	22	18.3	2.9
BRA12	Turnip kale	Wener Witte	0.9 ± 0.0	0.4	35.5	48.3	13.8	2
BRA13	White cabbage	Amager Polana	1.3 ± 0.0	0.1	30	45.6	22.8	1.5
BRA14	White mustard	-	0.9 ± 0.1	0	48	21.1	23.5	7.4
CUC1	Cucumber	Aladyn F1	0.8 ± 0.0	2	10.3	76.3	9.4	2
CUC2	Zucchini	Astra Polka	1.6 ± 0.2	0	7.6	86.5	5.8	0.1
CUC3	Pumpkin	Bambino	2.2 ± 0.1	0	4.4	92.3	3.1	0.2
CUC4	Pumpkin	Kabaczek Złoty Cepelin	0.9 ± 0.0	0.6	8.2	90.7	0.2	0.3
CUC5	Summer squash	Patison Polo F1	1.9 ± 0.0	0	3.3	93	3.4	0.3
FAB1	Alfalfa sprouting seeds	-	1.7 ± 0.1	0	3.3	11.3	85.4	0
FAB2	Beans for dry seeds	Borlotto lingua di fuoco nano	2.6 ± 0.0	1.3	86.3	12.5	0	0
FAB3	Broad bean	-	2.1 ± 0.1	1	7.5	85.5	6	0
FAB3	Dwarf bean	Esterka	1.4 ± 0.1	2.1	23.8	54.8	19.3	0
FAB5	Green lentil sprouting seeds	-	2.0 ± 0.1	0.8	8.8	79.4	10.7	0.3
FAB6	Mung bean sprouting seeds	-	2.6 ± 0.1	0.8	39.9	41.1	17.7	0.5
FAB7	Pea sprouting seeds	-	2.2 ± 0.1	1.8	14.8	73.6	9.2	0.6
POA1	Corn sprouting seeds	-	1.2 ± 0.0	0	10.9	84	3.6	1.5
SOL1	Eggplant	Black Beauty	1.6 ± 0.0	0	13.3	82.8	3.9	0
SOL2	Pepper	Kasia	3.4 ± 0.0	0	3.9	88.9	6.2	1
SOL3	Pepper	Ożarowska	1.7 ± 0.1	0	6.7	72.2	21.1	0
SOL4	Tomato	Betalux	1.4 ± 0.2	0.3	9.2	85.2	4.9	0.4
SOL5	Tomato	Krakus	1.6 ± 0.0	4	12.2	72.7	11.1	0
SOL6	Tomato	Malinowy Olbrzym	1.9 ± 0.1	0.2	9	81.3	9.5	0.3

Detection range of individual forms of phosphorus in the ^31^P NMR spectra of the neutralised extract of tested samples: P1—polyphosphates/diphosphates ((−15)–(−3.5) ppm); P2—phosphodiesters ((−1)–1.5 ppm); P3—phytates/orthophosphates (1.5–3.7 ppm); P4—other monoesters (3.7–6 ppm); P5—phosphonates (15–23 ppm).

**Table 2:** Common and botanical names of tested seeds, their taxonomic classification by families, estimated number of seeds per gram, and assigned symbols used in the text.

Symbol	Common Name	Variety of Seed	Botanical Name	Family	Seeds per Gram (s/g)
AMA1	Beetroot	Opolski	*Beta vulgaris* var. *Conditiva*	Amaranthaceae (AMA)	40–60
API1	Carrot	Dolanka	*Daucus carota*	Apiaceae (API)	600–700
API2	Celery	Talar	*Apium graveolens*		2500–2700
API3	Dill	Szmaragd	*Anethum graveolens*		700
API4	Foliate celery	Verde Pascal	*Apium graveolens* var. *dulce*		2500–2700
API5	Parsley	Alba	*Petroselinum crispum* convar. *Radicosum*		500–600
API6	Parsnip	Halflong White	*Pastinaca sativa*		200–220
AST1	Chicory	Palla Rossa 3	*Cichorium intybus* var. *foliosum*	Asteraceae (AST)	600–800
AST2	Sunflower sprouting seeds	-	*Helianthus annuus*		10–20
BRA1	Broccoli sprouting Seeds	*-*	*Brassica* *oleracea*	Brassicaceae (BRA)	315
BRA2	Brusselssprouts	Long Island	*Brassica oleracea* L. var. *gemmifera*		350–400
BRA3	Cauliflower	Beta	*Brassica oleracea* convar. *Botrytis*		300–400
BRA4	Chinese cabbage	Bristol	*Brassica pekinensis*		350–400
BRA5	Cress sprouting seeds	-	*Lepidium sativum*		400–420
BRA6	Kale sprouting seeds, fringed cabbage	-	*Brassica oleracea* L.		250–300
BRA7	Radish sprouting seeds	-	*Raphanus sativus* var. *sativus*		55
BRA8	Red cabbage sprouting seeds	-	*Brassica oleracea* var. *capitata rubra*		350
BRA9	Savoy cabbage	Langedijska	*Brassica oleracea* L. var. *sabauda*		350–400
BRA10	Swede (rutabaga)	Nadmorska	*Brassica napus*		350
BRA11	Turnip	Di milano a collettoviola	*Brassica rapa*		500
BRA12	Turnip kale	Wener Witte	*Brassica oleracea* var. *gongylodes*		300–350
BRA13	White cabbage	Amager Polana	*Brassica oleracea* var. *capitata alba*		300–350
BRA14	White mustard	-	*Sinapis* L.		150
CUC1	Cucumber	Aladyn F1	*Cucumis sativus*	Cucurbitaceae (CUC)	40–50
CUC2	Zucchini	Astra Polka	*Cucurbita pepo*		3–5
CUC3	Pumpkin	Bambino	*Cucurbita maxima*		2–3
CUC4	Pumpkin	Kabaczek Złoty Cepelin	*Cucurbita pepo*		3–5
CUC5	Summer squash	Patison Polo F1	*Cucurbita pepo*		6–8
FAB1	Alfalfa Sprouting seeds	-	*Medicago sativa* L.	Fabaceae (FAB)	470–500
FAB2	Beans for dry seeds	Borlotto lingua di fuoco nano	*Phaseolus vulgaris*		2–6
FAB3	Broad bean	-	*Vicia faba* L.		1
FAB3	Dwarf bean	Esterka	*Phaseolus vulgaris*		2–6
FAB5	Green lentil sprouting seeds	-	*Lens culinaris*		25
FAB6	Mung bean sprouting seeds	-	*Vigna radiata*		13
FAB7	Pea sprouting seeds	-	*Pisum sativum* L.		4–5
POA1	Corn sprouting seeds	-	*Zea mays* var. *saccharata*	Poaceae (POA)	8–10
SOL1	Eggplant	Black Beauty	*Solanum melongena*	Solanaceae (SOL)	200–250
SOL2	Pepper	Kasia	*Capsicum annuum*		170–200
SOL3	Pepper	Ożarowska	*Capsicum annuum*		170–200
SOL4	Tomato	Betalux	*Solanum lycopersicum* L.		250–270
SOL5	Tomato	Krakus	*Solanum lycopersicum* L.		250–270
SOL6	Tomato	Malinowy Olbrzym	*Solanum lycopersicum* L.		250–270

## Data Availability

On request to those interested.
